# EGFR/Src/Akt signaling modulates Sox2 expression and self-renewal of stem-like side-population cells in non-small cell lung cancer

**DOI:** 10.1186/1476-4598-11-73

**Published:** 2012-09-25

**Authors:** Sandeep Singh, Jose Trevino, Namrata Bora-Singhal, Domenico Coppola, Eric Haura, Soner Altiok, Srikumar P Chellappan

**Affiliations:** 1 Department of Tumor Biology, H. Lee Moffitt Cancer Center and Research Institute, 12902 Magnolia Drive, Tampa, FL, 33612, USA; 2 Department of Anatomic Pathology, H. Lee Moffitt Cancer Center and Research Institute, 12902 Magnolia Drive, Tampa, FL, 33612, USA; 3 Department of Thoracic Oncology, H. Lee Moffitt Cancer Center and Research Institute, 12902 Magnolia Drive, Tampa, FL, 33612, USA; 4 Current address: National Institute of Biomedical Genomics, 2nd floor, Netaji Subash Sanatorium, Kalyani, 741251, India; 5 Current address: Department of Surgery, University of Florida, 1600 SW Archer Rd, Rm 6175, Gainesville, FL, 32610-0109, USA

**Keywords:** Cancer stem-like cells, Side-population cells, Self-renewal, EGFR, Sox2

## Abstract

**Background:**

Cancer stem cells are thought to be responsible for the initiation and progression of cancers. In non-small cell lung cancers (NSCLCs), Hoechst 33342 dye effluxing side population (SP) cells are shown to have stem cell like properties. The oncogenic capacity of cancer stem-like cells is in part due to their ability to self-renew; however the mechanistic correlation between oncogenic pathways and self-renewal of cancer stem-like cells has remained elusive. Here we characterized the SP cells at the molecular level and evaluated its ability to generate tumors at the orthotopic site in the lung microenvironment. Further, we investigated if the self-renewal of SP cells is dependent on EGFR mediated signaling.

**Results:**

SP cells were detected and isolated from multiple NSCLC cell lines (H1650, H1975, A549), as well as primary human tumor explants grown in nude mice. SP cells demonstrated stem-like properties including ability to self-renew and grow as spheres; they were able to generate primary and metastatic tumors upon orthotopic implantation into the lung of SCID mice. In vitro study revealed elevated expression of stem cell associated markers like Oct4, Sox2 and Nanog as well as demonstrated intrinsic epithelial to mesenchymal transition features in SP cells. Further, we show that abrogation of EGFR, Src and Akt signaling through pharmacological or genetic inhibitors suppresses the self-renewal growth and expansion of SP-cells and resulted in specific downregulation of Sox2 protein expression. siRNA mediated depletion of Sox2 significantly blocked the SP phenotype as well as its self-renewal capacity; whereas other transcription factors like Oct4 and Nanog played a relatively lesser role in regulating self-renewal. Interestingly, Sox2 was elevated in metastatic foci of human NSCLC samples.

**Conclusions:**

Our findings suggest that Sox2 is a novel target of EGFR-Src-Akt signaling in NSCLCs that modulates self-renewal and expansion of stem-like cells from NSCLC. Therefore, the outcome of the EGFR-Src-Akt targeted therapy may rely upon the expression and function of Sox2 within the NSCLC-CSCs.

## Introduction

Despite significant therapeutic advances, lung cancer causes the maximum number of cancer related deaths worldwide [1]. In the United States, ~85% of the patients diagnosed with NSCLCs, die within five years [2-4], thus, highlight a need for better understanding of the cellular and molecular events underlying the genesis of this disease. Cancer stem cell model has emerged as a viable explanation for the initiation and progression of the aggressive cancers like NSCLCs.

Cancer stem cell model suggests that cancer stem-like cells (CSCs) are a subpopulation of cells within the tumor that have the deregulated properties of normal stem cells with sustained self-renewal, and can generate secondary tumors that recapitulate the heterogeneity and diversity of original tumor [5-8]. CSCs are considered to be responsible for tumor initiation, propagation, recurrence and resistance to therapy [9,10]. Hoechst 33342 dye excluding cells, termed side-population (SP) cells, have been described as CSCs in a variety of tumor types, including NSCLCs [11], where they have been shown to display increased tumorigenicity when transplanted into immunocompromised mice [12,13] as compared to major population (MP) cells. SP phenotype is dependent on the differential ability of cells to efflux the Hoechst 33342 dye via the ATP-binding cassette (ABC) family of transporter protein, mainly ABCG2 (breast cancer resistance protein, BRCP1) which is specifically expressed on the cell membrane of stem cell populations [14]. Earlier studies have demonstrated the existence of SP cells in various established human NSCLC cell lines [11] but their ability to generate tumors in lung microenvironment as well as the signaling pathways governing their stem-like properties remain to be elucidated.

The transcription factors Oct4, Sox2 and Nanog have been identified as core regulators that maintain the self-renewal of embryonic stem cells [15]. These factors are overexpressed in various cancers and are associated with malignant progression and poor prognosis including NSCLCs [16,17], suggesting that the core regulators that govern normal stem cell self-renewal may also maintain the stem-like properties of CSCs in cancers. However, the influence of NSCLC specific oncogenic pathways on the expression of these factors remains relatively unknown. Alterations in EGFR-gene like copy number gains and/or mutant allele-specific amplifications are associated with NSCLC pathogenesis. In addition, activation of EGFR signaling increases the self-renewal capacity of neural precursor cells and brain tumor stem cells [18-20]**.** In this study, we provide biochemical and biological evidence that SP cells isolated from established human NSCLC cell lines and tumors are highly enriched in NSCLC-CSCs and EGFR-Src-Akt signaling axis contributes significantly to the self-renewal of SP cells. Interestingly, Sox2 transcription factor is the predominant downstream target of EGFR signaling in these cells and plays a major role in self-renewal growth and expansion of SP cells, independent of Oct4 and Nanog.

## Results

### SP cells are enriched with tumorigenic cells and produce highly invasive tumors

**Figure 1:**
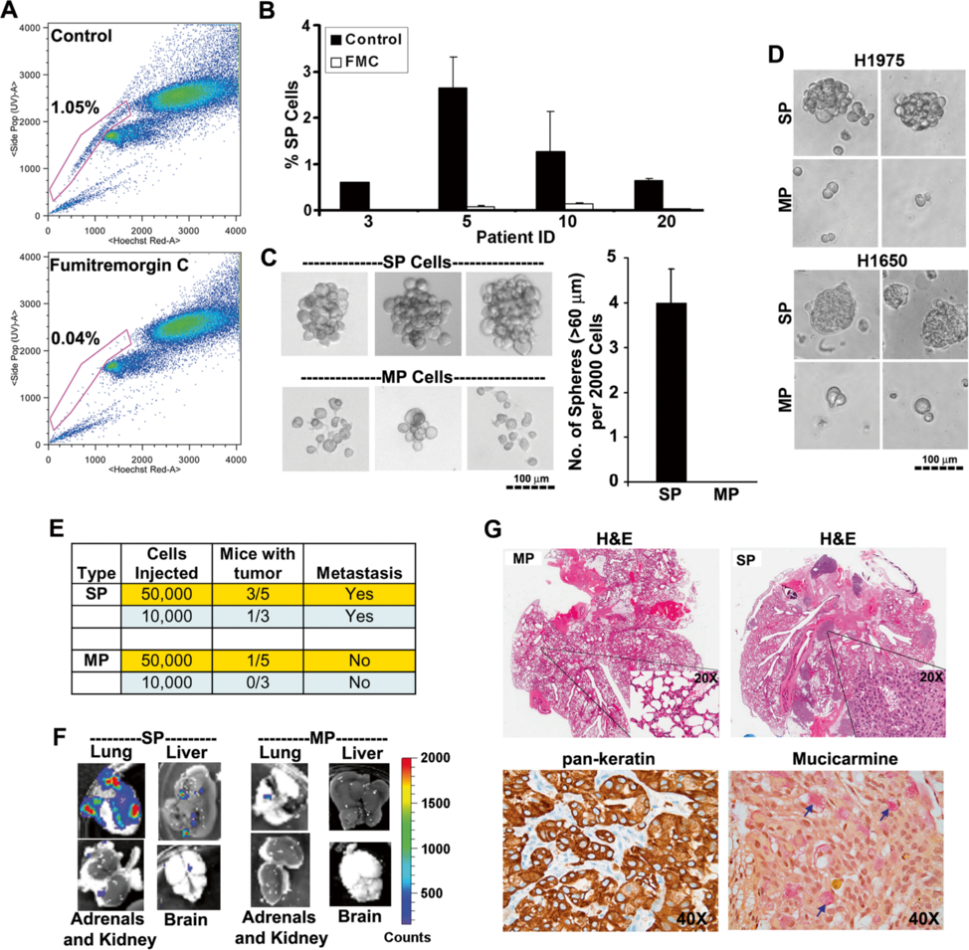
**SP cells exhibit stem-like properties.** (**A**) FACS analysis on single cell suspension of human NSCLC xenograft stained with Hoechst 33342 dye showing SP cells. Fumitremorgin C (FTC) inhibited the efflux of the dye and caused the disappearance of SP cells. (**B**) SP cell frequency in presence or absence of FTC in four different human tumor xenografts. (**C**) Sphere formation assay on SP or MP cells grown in stem cell selective media for 10 days. The pictures of representative spheres are presented. The bar diagram show the average (±SD) number of spheres formed from 2000 cells. (**D**) SP or MP cells from H1650 and H1975 cell lines were plated in serum free medium supplemented with EGF and bFGF for 10 days under self-renewal assay conditions. The pictures of representative spheres are depicted (**E**) Indicated number of SP and MP cells from A549-Luc cell line were implanted into the right lung tissue of SCID mice. The tumor incidence and metastasis was monitored for 12 weeks by bioluminescence imaging. (**F**) Ex-vivo images of the lung, liver, kidney/adrenals and brain captured at the end of experiment. Metastasis was prevalent in SP cells implanted mice. (**G**) H&E, pan-keratin (brown staining) and mucicarmine (pink staining, indicated by arrow head) staining of whole lungs from mice implanted with SP or MP cells.

In an attempt to identify NSCLC stem-like cells, SP analysis was conducted on four primary human NSCLC explants grown in athymic nude mice. SP cells appeared as a well separated population as described previously [21]. As shown in Figure 1A, a specific inhibitor of ABCG2 [22], Fumitremorgin C (FTC) could block the appearance of SP phenotype. All the four tumor samples displayed the presence of SP cells with varying frequency ranging from 0.6-3%, and could be significantly blocked by FTC (Figure 1B).

Self-renewing normal or cancer-stem-like cells can be expanded as non-adherent spheres when cultured at low density in serum free, stem cell selective medium; differentiated cells do not grow or form spheres under these conditions [6,23,24]. The self-renewal property of SP cells was examined by performing sphere formation assay on sorted SP and MP cells isolated from human tumor xenografts. While sorted SP cells were able to grow as spheres, MP cells had markedly less capacity to grow under identical conditions (Figure 1C).

Attempts were then made to assess the presence of SP cells in human NSCLC cell lines. As shown in later sections, A549 (K-Ras mutant; wild type EGFR amplification), H1650 (EGFR mutant; Exon 20 deletion: delE746-A750) and H1975 (EGFR mutant; L858R and T790M mutations), contained SP-cells with varying frequency. Appearance of SP cells was completely blocked by FTC. Sorted SP cells were able to grow as spheres whereas MP cells showed markedly reduced capability (Figure 1D) suggesting that NSCLC-SP cells are enriched with CSCs.

The stem cell like property of NSCLC-SP cells was verified by evaluating its ability to form tumors in the lung microenvironment. Sorted SP and MP cells from A549 cells stably expressing the luciferase gene were orthotopically implanted into the left lung of SCID mice and tumor growth was monitored for 12 weeks. As shown in Figure 1E, SP cells generated primary tumors in the lung more efficiently than MP cells. At the end of the experiment, lungs, liver, kidney and brain were excised from each mouse and ex-vivo images were examined for the presence of metastasized luciferase positive cells. Mice injected with SP cells demonstrated substantial tumor burden throughout the lungs and showed luminescent metastatic loci in liver, kidney (adrenals) and brain (Figure 1F). In contrast, MP cells formed only one luminescent focus in the lung of one mouse (out of five) injected with 50 000 MP cells and there was no metastasis (Figure 1F). These results were confirmed by H&E staining; further, tumors formed within the lung from SP cells, recapitulated the histopathology of adenocarcinoma as confirmed by positive staining with pan-keratin antibody as well as mucicarmine dye (Figure 1G). These data suggested that SP cells are enriched with tumorigenic cells and can develop metastatic tumors *in vivo*.

### SP cells exhibit molecular markers of stem-like cells

**Figure 2:**
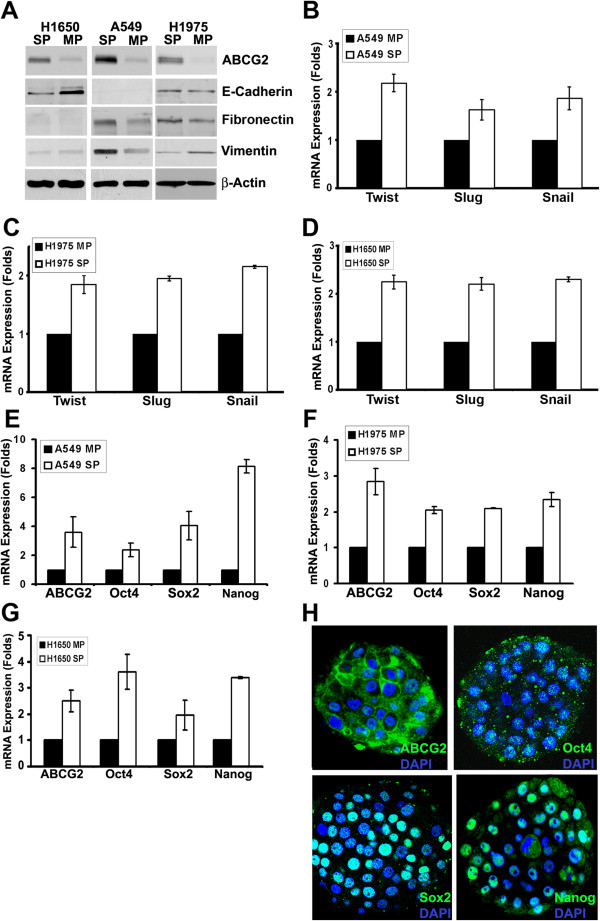
**SP cells express stem cell-like markers.** (**A**) Western blot analysis on SP and MP cells from A549, H1650 and H1975 cell lines for the indicated proteins. β-Actin was used as the loading control. (**B**, **C**, **D**) SP and MP cells were analyzed by real time qRT-PCR method for the expression of Twist, Slug and Snail genes. Average (±SD) fold change between MP and SP cells were plotted. (**E**, **F**, **G**) SP and MP cells were analyzed by real time qPCR method for the expression of ABCG2, Oct4, Sox2 and Nanog genes. Average (±SD) fold change between MP and SP cells were plotted. (**H**) The spheres formed in self-renewal assays of H1650 SP cells were stained for the expression of ABCG2, Oct4, Sox2 and Nanog. Confocal image of one of the z-stacks for a sphere is presented. DAPI was used to stain the nucleus.

Recent reports suggest that epithelial cells acquire cancer stem cell properties upon induction of epithelial to mesenchymal transition (EMT) [25]. To evaluate whether SP cells show features of EMT, SP and MP cells from A549, H1650 and H1975 were examined for the levels of EMT markers like E-cadherin, Vimentin and Fibronectin. As shown in Figure 2A, ABCG2 expression was significantly higher in the SP fraction in all the three cell lines. The levels of E-cadherin was lower in H1650-SP cells as compared to MP cells, however, it was undetectable in A549 and unchanged in H1975 cells. Fibronectin was detected at higher levels in A549 and H1975-SP cells, but undetectable in H1650 cells. Vimentin level was higher in A549-SP cells, but low in H1975 and H1650 SP cells. Although the levels vary in a cell type dependent manner, these results suggest that, SP cells express proteins indicative of EMT without any external stimuli to the cells (Figure 2A). The molecular basis for the differential expression of the EMT markers was then examined. Transcription factors like Twist, Slug and Snail have been demonstrated to be capable of coordinating the EMT program during embryonic development and in cancers [26,27]. Therefore, we next assessed the expression of these transcription factors in SP and MP cells. Real-time PCR analysis revealed that Twist, Slug and Snail transcription factors are expressed at higher levels in SP cells in all the three NSCLC cell lines (Figure 2B, C, D).

The expression of Oct4, Sox2 and Nanog transcription factors was next examined in SP cells. Real-time PCR analysis showed elevated levels of ABCG2, Oct4, Sox2, and Nanog in the SP fraction in all the three cell lines. (Figure 2E, F, G). Further, SP cells from H1650 cells growing as spheres showed expression of ABCG2, Oct4, Sox2 and Nanog proteins by fluorescence microscopy (Figure 2H), indicating the undifferentiated growth of self-renewing SP cells within the spheres.

### EGFR tyrosine kinase inhibitors downregulate self-renewal and SP phenotype

**Figure 3:**
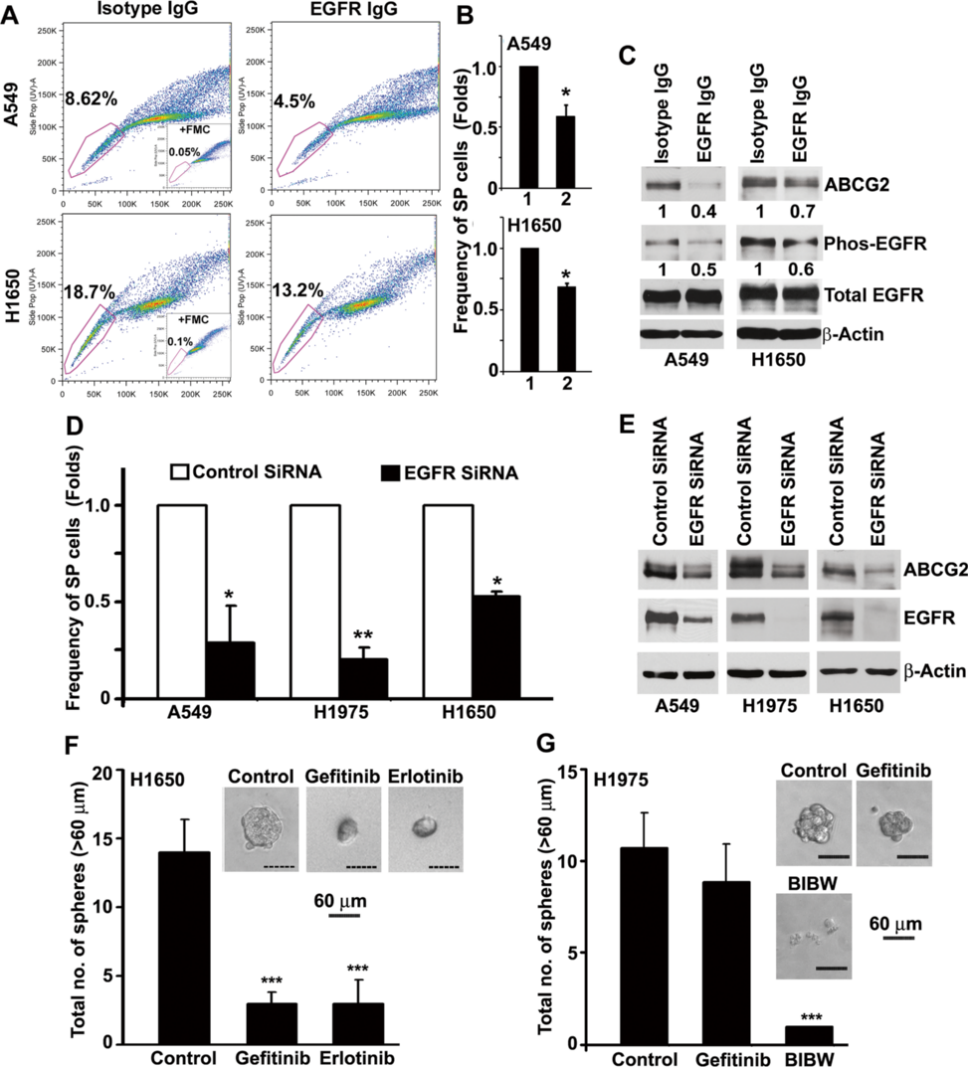
**Inhibition of EGFR suppresses SP frequency and self-renewal.** (**A**) H1650 and A549 cells revealed a significant decrease in SP frequency after treatment with 10 μg/ml EGFR-neutralizing antibodies for 5 days. (**B**) Frequency of SP cells in control antibody (1) and EGFR-neutralizing antibodies (2) treated cells. (**C**) Western blot analysis for ABCG2 and phospho-EGFR after EGFR-neutralizing antibody treatment. (**D**) SP frequency in NSCLC cells transfected with siRNA against EGFR or a control siRNA. (**E**) Expression of ABCG2 and EGFR in antibody treated cells. (**F** and **G**) SP cells were sorted and plated for self-renewal assay in the presence or absence of indicated drugs. Average number of spheres generated per well from 1000 cells is plotted (mean ± SD). Phase contrast microscopy images of the spheres in presence or absence of drugs are presented. * represent the p value of <0.05; ** represent the p value of <0.01; *** represent the p value of <0.001.

Experiments were conducted to explore the molecular mechanisms involved in the self-renewal of SP cells. Since aberrant EGFR signaling is implicated with the initiation and progression of lung cancer, we first assessed SP frequency and expression of ABCG2 in the presence of an antagonistic antibody against EGFR. Cells were mixed with 10 μg/ml anti-EGFR antibody or an isotype control and plated in 2% FBS containing media for 5 days. Blocking EGF-receptors resulted in a significant decrease in SP frequency in both A549 and H1650 cells (Figure 3A, B), along with decreased EGFR phosphorylation as well as ABCG2 expression in both the cell lines (Figure 3C). Confirming these results, depletion of EGFR expression by a siRNA resulted in decreased SP frequency and ABCG2 expression in A549, H1650 and H1975 cells (Figure 3D, E).

To further evaluate whether EGFR signaling contributed to the self-renewal property of H1650 SP cells, sphere formation assay was conducted in the presence or absence of EGFR inhibitors Gefitinib or Erlotinib. As shown in Figure 3F, inhibition of EGFR-kinase activity by 500 nM of Gefitinib or Erlotinib, demonstrated a 5–7 fold (p < 0.001) decrease in the number of spheres; further the size of the spheres was also significantly reduced.

A secondary point mutation in exon 20 of EGFR (T790M) is associated with acquired resistance to gefitinib or Erlotinib, but this can be overcome by the irreversible EGFR-tyrosine kinase inhibitor BIBW2992 (BIBW). We tested the effect of 500 nM of gefitinib and 200 nM of BIBW on EGFR phosphorylation and self-renewal growth of SP cells from H1975 cell line, which harbors gefitinib-resistant-T790M mutation along with Gefitinib responsive-L858R mutation in exon 21. Western blot analysis showed that tyrosine phosphorylation of EGFR was insensitive to 500 nM concentration of gefitinib, whereas significant downregulation occurred after treatment with 200 nM of BIBW in H1975 cells (Additional file 1: Figure S1). Consistent with this, BIBW could significantly inhibit the self-renewal of SP cells from H1975 cells (Figure 3G).

### Adherent cultures of SP cells maintain stem-like properties

**Figure 4:**
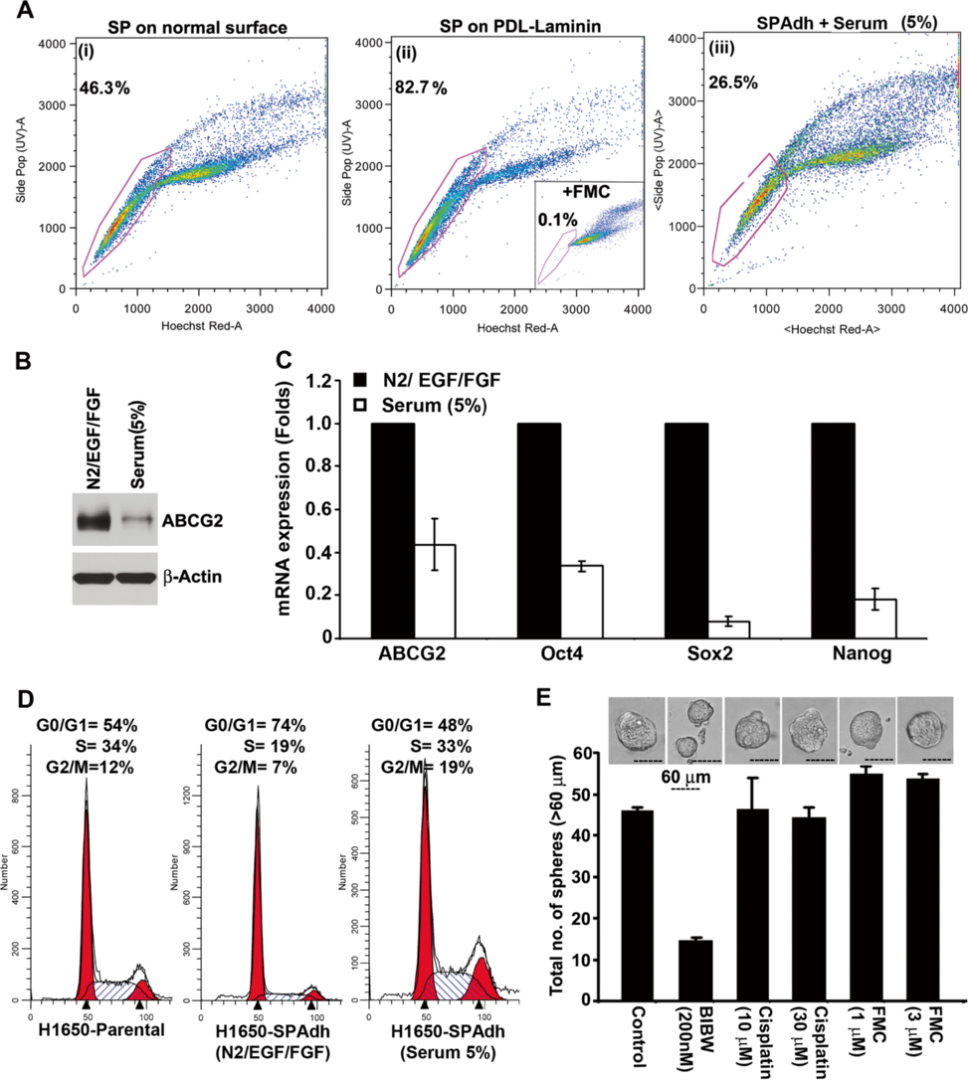
**Establishment and characterization of H1650-SPAdh cells.** (**A**) SP cells from H1650 cell line was plated on normal tissue culture plate (i) or poly-D-lysine-laminin (PDL-Laminin) coated surface (ii) in serum free medium containing N2-supplement, EGF and bFGF. H1650-SPAdh cells growing in self-renewing condition was cultured in serum for 5 days to induce differentiation, and reanalyzed for SP frequency (iii). (**B**) Serum induces differentiation of SPAdh cells as seen by ABCG2 expression and (**C**) real time qPCR analysis for stem cell markers, *ABCG2, Oct4, Sox2, Nanog*. (**D**) Cell cycle analysis for parental-H1650 or H1650-SPAdh and serum differentiated H1650-SPAdh cells grown on PDL-Laminin coated surface. Histograms were plotted using ModFit program. (**E**) The average number of spheres generated from 1000 H1650-SPAdh cells is plotted (mean ± SD). Phase contrast microscopy images of the spheres in presence or absence of indicated drugs.

To conduct further molecular studies on SP cells, we attempted to establish adherent cell cultures of isolated SP cells from A549, H1975 and H1650 cell lines, as suggested for glioma stem cells [28]. Isolated SP cells were plated on uncoated or Poly-D Lysine + Laminin coated culture plates in serum free, stem cell media. While A549-SP and H1975-SP cells detached from the surface, H1650-SP cells grew as an adherent culture. As shown in Figure 3A, H1650-SP cells cultured on uncoated surface failed to maintain SP phenotype with high frequency (Figure 4A, (i)), but 80% of the cells maintained as SP cells even after 5 passages when plated on PDL + laminin coated surface (Figure 4A, (ii); H1650-SPAdh cells). H1650-SPAdh cells cultured back in 5% FBS containing medium for 10 days could recapitulate the proportion of SP and MP cells found in parental H1650 cells (Figure 4A, (iii)), with a concomitant reduction in expression of ABCG2 (Figure 4B), as well as Oct4, Sox2 and Nanog mRNA as seen by R-PCR (Figure 4C). Cell cycle analysis showed that H1650-SPAdh cells were slow cycling compared to parental cells (Figure 4D), having approximately 20% higher number of cells in G_0_/G_1_ phase; but upon serum-induced differentiation, H1650-SPAdh cells acquired cell-cycle phase distribution comparable to H1650-parental cells (Figure 4D).

Treatment of H1650-SPAdh cells with 200 nM BIBW significantly suppressed the number as well as the size of spheres (Figure 4E); at the same time, treatment with 30 μM cisplatin did not affect the number or the size of the spheres formed by H1650-SP cells, suggesting enhanced chemoresistance of these cells. Further, the sphere-formation ability of SP was not altered by the ABCG2 inhibitor, FTC, suggesting that self-renewal of SP cells was independent of ABCG2 activity (Figure 4E).

### Inhibition of EGFR-Src-Akt signaling downregulates Sox2 expression

**Figure 5:**
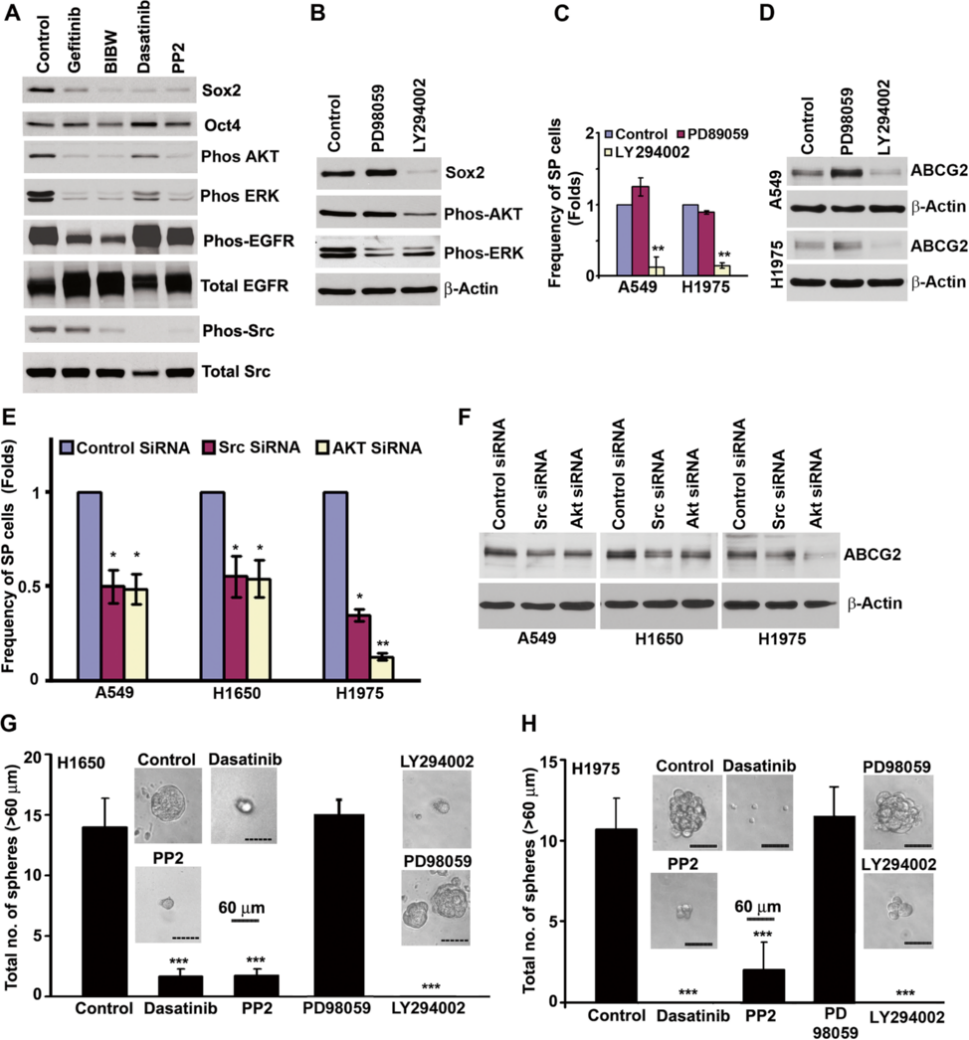
**Inhibition of EGFR signaling downregulates Sox2 expression (A) H1650-SPAdh cells were treated with EGFR or Src inhibitors for 4 days and western blot analysis was performed for the indicated proteins.** (**B**) H1650-SPAdh cells were treated with MEK or PI3K inhibitor for 4 days and levels of Sox2 and activation of Akt or ERK was evaluated by western blot. (**C**) A549 and H1975 cells were treated with MEK or PI3K inhibitor for 5 days and the frequency of SP cells were examined by SP analysis. Data represents the fold change in mean (± SD) of SP frequency. (**D**) ABCG2 expression upon inhibitor treatment as detected by western blotting. (**E**) SP cell frequency in NSCLC cell lines transfected with specific siRNAs against Src or AKT. (**F**) ABCG2 expression in siRNA transfected cells as detected by western blotting. (**G** and **H**) SP cells were sorted and plated for self-renewal assay in the presence or absence of indicated drugs. Average number of spheres generated from 1000 cells is plotted (mean ± SD). Phase contrast microscopy images of the spheres in presence or absence of drugs are presented. * represent the p value of <0.05; ** represent the p value of <0.01; *** represent the p value of <0.001.

Experiments were conducted to examine the downstream signaling events from EGFR that modulates self-renewal of SP cells and whether these pathways impinge transcription factors associated with stemness. Role of c-Src in the process was first examined since Src is altered in NSCLC [29,30]. H1650-SPAdh cells were treated with EGFR or Src TKIs and the levels of Oct4 and Sox2 was assessed by western blotting (Figure 5A). EGFR inhibition by 500 nM gefitinib or 200 nM BIBW as well as inhibition of Src activity by 200 nM dasatinib or 1 μM PP2 markedly reduced Sox2 expression; Oct4 level was not affected (Figure 5A). These results were verified by immunoflorescence experiments. Similar to Oct4, there was no significant difference in Nanog expression; however, the number Sox2 positive cells were significantly decreased in response to the treatment of EGFR- and Src-TKIs (Additional file 1: Figure S2).

Inhibition of EGFR as well as Src signaling resulted in decreased phosphorylation of EGFR, Src, ERK and Akt (Figure 5A). Contribution of ERK and Akt pathways to EGFR mediated induction of Sox2 was next examined in H1650SPAdh cells. Phosphorylation of ERK was suppressed by MEK inhibitor PD98059 and AKT-phosphorylation was suppressed by the PI3-kinase inhibitor, LY294002. However, PI3-Kinase inhibited H1650SPAdh cells also resulted in slight inhibition in ERK phosphorylation (Figure 5B). A similar observation has been reported in earlier studies where PI3-Kinase signaling was demonstrated to regulate the ERK phosphorylation in T-cell-receptor (TCR) signaling [31] and PDGFR mediated signaling [32]. However, as shown in Figure 5B, inhibition of MEK activity did not affect the levels of Sox2 while the PI3-kinase inhibition, markedly reduced its levels with corresponding reduction in SP frequency (Figure 5C) and ABCG2 expression (Figure 5D). These results were confirmed using siRNAs to Src and Akt. As shown in Figure 5E, SP frequency was significantly downregulated in both Akt and Src siRNA transfected A549, H1650 and H1975 cells as compared to the control siRNA transfected cells, with a corresponding reduction in ABCG2 expression (Figure 5F). Similar inhibitory effects were observed upon silencing of two other Src family members, Fyn and Yes (data not shown).

To determine whether Src or Akt signaling facilitates self-renewal of SP cells, sphere formation assay was conducted on SP cells in presence or absence of Src inhibitors Dasatinib or PP2, MEK inhibitor PD98059 as well as Akt inhibitor LY294002. As shown in Figures 5G and 5H, Src-kinase inhibitors dasatinib or PP2, as well as PI3K/Akt inhibitor LY294002 showed a significant (5–7 fold; p < 0.001) decrease in sphere formation; MEK inhibition by PD98059 did not have any significant effect on self-renewal. The average size of the spheres formed was found to be 7–10 folds smaller than the untreated cells. Collectively, these data indicated that inhibition of EGFR/Src/Akt signaling results in depletion of Sox2 expression and decreased self-renewal of SP cells.

### Suppression of Sox2 expression is sufficient to inhibit the self-renewal of SP cells

**Figure 6:**
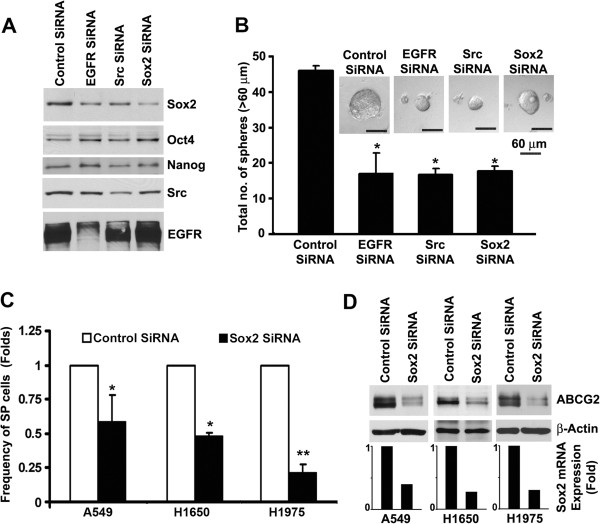
**Sox2 is necessary for maintaining the self-renewal of SP cells.** (**A**) H1650-SPAdh cells were transfected with non-targeting control siRNA, or siRNA against EGFR, Src, or Sox2 and western blot analysis was performed for Sox2, Oct4, EGFR and Src expression. (**B**) siRNA transfected cells were plated for self-renewal assay and analyzed after 5 days of plating. Average number of spheres generated per well from 1000 cells is plotted (mean ± SD). (**C**) NSCLC cell lines were transfected with control or Sox2 siRNAs and SP frequency was evaluated. Average fold change in SP-frequency (± SD) is plotted. (**D**) Decrease in ABCG2 expression in Sox2 siRNA transfected cells was detected by western blotting. The Sox2 expression was evaluated by qRT-PCR and results are presented as bar diagram.

Since inhibition of EGFR/Src/Akt signaling specifically downregulated the expression of Sox2, we examined the contribution of Sox2 to the self-renewal of H165SP-Adh cells. Transient transfection of EGFR and Src siRNA in H1650-SPadh cells reduced EGFR expression by 60% and Src expression by 50%. Reduction in EGFR or Src expression decreased the levels of Sox2 by 50% and 40% respectively; the expression of Oct4 and Nanog was not altered (Figure 6A). In addition, depletion of EGFR or Src by siRNA suppressed the sphere formation by 2–3 folds (Figure 6B)**.** To further explore the function of Sox2 in self-renewal of SP cells, we depleted Sox2 expression in H1650-SPadh cells. Transient transfection of Sox2 siRNA reduced the expression of Sox2 by 60% (Figure 6A). Depletion of Sox2 expression did not significantly alter the expression of Oct4 or Nanog expression in H1650-SPadh cells (Figure 6AB), and reduced the sphere formation by approximately 2.5 folds (Figure 6) with a corresponding reduction in the average size (p = 0.003). Depletion of Sox2 expression resulted in a pronounced decrease in the frequency of SP cells (Figure 6C) as well as ABCG2 expression (Figure 6D) in A549, H1650 and H1975 cells compared to control siRNA transfected cells. Similar results were obtained when a different siRNA to Sox2 was used (Additional file 1: Figure S3). Collectively, these results suggest that Sox2 gene has a direct role in maintaining cancer stem cell characteristics and self-renewal of SP cells from NSCLC.

### Sox2 is expressed in NSCLC and is associated with metastatic progression

**Figure 7:**
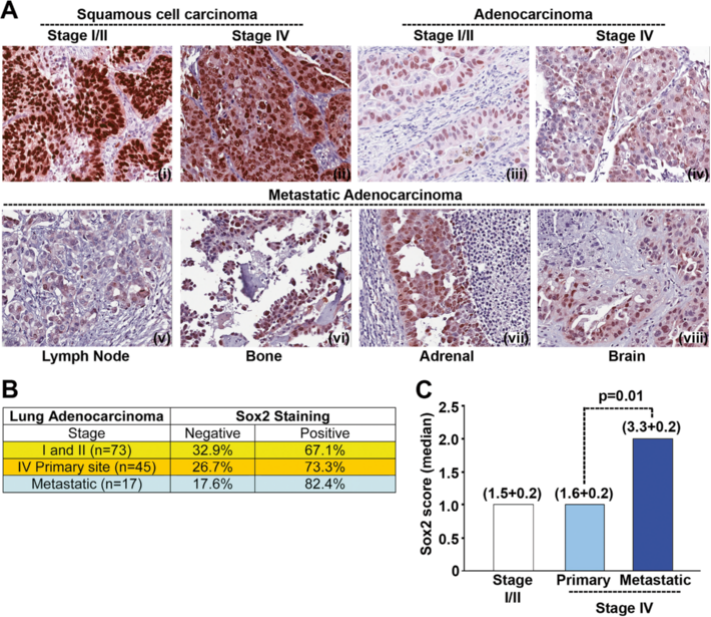
**Sox2 expression correlates with metastatic progression of adenocarcinoma.** (**A**) Sox2 expression in Lung squamous cells carcinoma, adenocarcinoma and metastatic adenocarcinoma was analyzed from stage I/II or stage IV patients samples by immunohistochemistry. (**B**) Semi-quantitative scoring was performed for adenocarcinoma samples and the tumors with score of one or more was considered positive for Sox2 expression and listed. (**C**) Median score for Sox2 expression was calculated and plotted for different stages of NSCLC progression. The mean (± SD) of score is mentioned in parenthesis. Metastatic tumors showed significantly (*p = 0.01*) higher expression of Sox2.

Our data showing that depletion of Sox2 affects the self-renewal properties of stem-like cells, we next examined Sox2 expression in a panel of NSCLC tumor samples obtained from stage I/II or stage IV patients on tissue microarrays (TMAs) by immunohistochemistry. Samples from 193 patients with NSCLC-stage I/II disease including 73 with adenocarcinoma were on one TMA; samples from 103 stage IV-NSCLC patients including 45 with adenocarcinoma from primary site and 17 adenocarcinoma samples from the metastatic sites were on the second TMA. In accordance with earlier reports, Sox2 was strongly expressed in squamous cell carcinoma (SCC) samples for both stage I/II and IV patients (Figure 7A (i and ii)). In contrast to SCCs, adenocarcinoma samples had significantly lower expression of Sox2. Sox2 positive cells were heterogeneously distributed in adenocarcinoma samples for both stage I/II and IV patients (Figure 7A (iii and iv). While there was no significant difference in Sox2 expression between different grades of tumors, elevated expression of Sox2 was positively associated with metastatic progression. Representative images for adenocarcinoma-metastases are shown in Figure 7A (v-viii). Approximately 67% of stage I/II (n = 73) and 73% of stage IV (primary site, n = 45) tumors were detected as positive for Sox2 expression (score ≥ 1) using a semi-quantitative scoring system. Compared to the primary site tumor for stage IV patients, higher numbers of metastasized tumors were positive for Sox2 (Figure 7B). The median score for Sox2 expression is represented as histogram (Figure 7C). The average score for Sox2 expression was found to be significantly higher (*p* = 0.01) in metastasized tumors as compared to the primary site or lower stage tumors. Overall, Sox2 was expressed in all stages of adenocarcinoma and its levels were significantly higher in metastatic lesions.

## Discussion

In the current study, we used the SP phenotype to identify and enrich a subpopulation of NSCLCs with the properties ascribed to CSCs. The studies presented here demonstrates a specific and significant role for EGFR signaling cascade in facilitating the self-renewal growth and expansion of the side population cells from NSCLCs.

Our study, in accordance with earlier studies [11], [33], confirmed the presence of SP cells in established human NSCLC cell lines and in human tumor xenografts with the properties of CSCs. Comparing the self-renewal ability of SP and MP cells isolated from human tumor xenografts, we found that approximately 0.2% SP cells were able to self-renew and form spheres, whereas MP cells were unable to self-renew. Comparing the percentage of sphere forming cells in SP cells, we estimate that approximately 1-2% of SP cells from established cell lines may have stem-like properties; therefore, SP phenotype may not be the exclusive marker for CSCs, but can be used to enrich stem-like cells from NSCLCs.

SP cells were found to be more tumorigenic *in vivo*, confirming the enrichment of tumor initiating cells in SP compartment. These cells were able to produce highly invasive disease upon implantation into the lungs. Also, the direct association of stem-like cells with generation of metastatic disease may be supported by our observation where a significant correlation was observed between high Sox2 expressions in the metastatic tumors of lung adenocarcinoma patients. Recent reports indicate that the normal epithelial cells acquire the CSCs properties upon induction of EMT governed by various cytokines and growth factors from stromal cells [10,25]. Our results demonstrate that SP-cells intrinsically exhibit loss of epithelial markers and/or the gain of mesenchymal markers as compared to MP cells and could be due to the higher expression of transcription factors Twist, Slug and Snail, which are known to be involved in maintaining the mesenchymal phenotype. Together with the expression of embryonic stem cell transcription factors like Oct4, Sox2, and Nanog along with the exhibition of EMT like features and orthotopic tumor-forming ability, collectively suggest that SP cells isolated from NSCLC cell lines and tumors have stem-like properties. The observation that EGFR signaling affects stem-like functions of SP cells is intriguing, given that several EGFR tyrosine-kinase inhibitors have efficacy against NSCLCs [34,35]. Interestingly, EGFR appears to regulate Sox2 levels, through the Src-Akt pathway; Sox2 has been shown to be regulated by Akt in ES cells, through the inhibition of proteasomal degradation [36]. Consistent with these results, our observation suggest that inhibition of EGFR-Src-Akt signaling downregulates Sox2 levels along with a reduction in ABCG2 levels. This decrease in ABCG2 expression upon EGFR inhibition is probably a causal effect of Sox2 depletion-mediated differentiation of SP into MP cells.

The fact that EGFR-pathway inhibition resulted in specific depletion of Sox2 without any significant effect on Oct4 or Nanog expression suggests that their expression may be regulated through independent mechanisms in NSCLC SP cells. Our results as well as an earlier report [37] suggest that Sox2 is expressed in both low as well as high stage adenocarcinomas irrespective of their grades. However, Oct4 or Nanog expression was found to be associated only with the high grade lung adenocarcinoma and not expressed in low grade tumors [17,37]. Therefore, we predict that the EGFR pathway inhibition may exert its favorable effects only for those tumors where Sox2 is the major determinant in controlling the self-renewal of CSCs. Interestingly, a recent study showed that the ectopic overexpression of Oct4 and Nanog increases the tumor initiating property of A549 cells [17]. In agreement with these reports, we find that specific and independent depletion of Oct4 or Nanog also resulted in decrease in SP phenotype but in a cell type dependent fashion (Data not shown). Two recent reports demonstrate that ectopic expression of Sox2 increased the frequency of side population cells and tumor formation in mouse and human NSCLC cell lines [33,38]. These reports strongly suggest that Sox2 expressing cells harbor the stem cell-like properties. Our observation further strengthens this postulation where we demonstrate that Sox2 depletion was sufficient to inhibit the self-renewing property SP cells in all the three NSCLC cell lines.

In addition to the mutation in EGFR signaling, perturbation of p53 activity is another important event occurs in initiation and progression of NSCLCs [39]. Recently, p53 is shown to have certain roles in promoting the differentiation of human embryonic stem cell through repression of factors like Oct4, Klf4, Lin28A, and Sox2 [40]. However, there is not much information available on the direct role of p53 transcriptional activities in regulating Sox2 expression in stem-like cells in cancer, and would be interesting to explore in future.

## Conclusions

**Figure 8:**
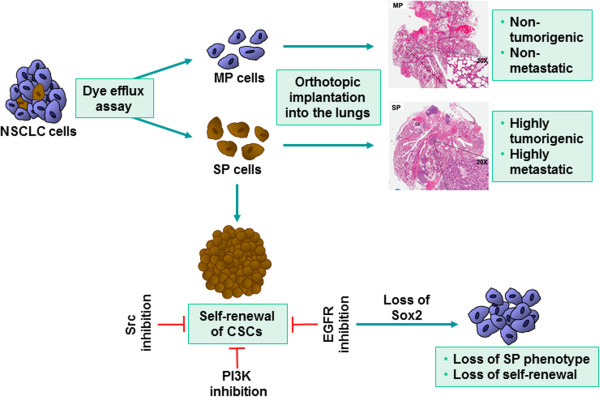
**A schematic depiction of the signaling events that regulate the biology of SP cells.** Isolated SP cells form metastatic tumors but MP cells do not. Inhibiting EGFR, Src or PI3-kinase significantly impairs the self-renewal properties of SP cells.

Figure 8 summarizes the role of Sox2 in SP cell biology and tumor growth. While certain frequency of isolated SP cells from NSCLC exhibit stem cell-like properties and can form metastatic tumors, more differentiated MP cells are greatly impaired in their ability to generate tumors. Further, inhibition of EGFR pathway including Src and PI3-kinase could strongly inhibit the expression of Sox2, suppressing the self-renewal properties of SP cells. Therefore, relative Sox2 expression and functions within the tumor-CSCs may be a major determinant in EGFR-targeted therapy against NSCLCs. This information might also be potentially useful to overcome the acquired resistance to EGFR therapies, by targeting downstream targets of EGFR signaling, including Sox2. Additional investigations in this direction might lead to the development of more effective therapeutic agents to combat NSCLC, especially those harboring EGFR mutations.

## Materials and methods

### Cell lines and tumor samples

H1650, and H1975 cell lines were obtained from ATCC and maintained in RPMI or DMEM containing10% fetal bovine serum (FBS; Mediatech) in 5% CO_2_ at 37°C. Human tumor xenografts were obtained from SA laboratory.

### Inhibitors, siRNAs and antibodies

Gefitinib, Erlotinib, BIBW2992 and Dasatinib were purchased from LC laboratories. PP2 and Fumitremorgin C (FTC) were purchased from Sigma Inc. In the present study, Gefitinib or erlotinib is used at 500 nM, dasatinib or BIBW2992 is used at 200 nM and PP2 is used at 1 μM dose. siRNA against EGFR, Src family kinases, Akt and Sox2, Oct4 and Nanog was purchased from Santa Cruz Biotechnology or OriGene Technology Inc. Primary antibodies against Sox2 (#3579), Oct4 (#2750), Nanog (#4903), Phos-Src-pY^416^ (#2101), pERK1/2 (#4376) and phospho-AKT-pS^473^ (#4058) were purchased from Cell Signaling Technology; Phos-EGFR-pY^1068^ (#44788G) from Invitrogen; EGFR neutralizing antibody (#05-101) from Milipore and isotype matched mouse IgG were purchased from Biolegend.

### RNA preparation and qRT PCR analysis

RNA preparation and RT-PCR analysis was performed as described earlier [41]. Fold inductions were calculated using the formula 2^–(ddCt)^ using GAPDH as internal control gene. The gene-specific primer pairs were as follows. ABCG2 (F) 5’-CAC AAG GAA ACA CCA ATG GCT-3’, ABCG2 (R) 5’-ACA GCT CCT TCA GTA AAT GCC TTC-3’; Oct4 (F) 5’-ACA TCA AAG CTC TGC AGA AAG AAC-3’, Oct4 (R) 5’-CTG AAT ACC TTC CCA AAT AGA ACC C-3’, Sox2 (F) 5’-GGG AAA TGG GAG GGG TGC AAA AGA-3’, Sox2 (R) 5’-TTG CGT GAG TGT GGA TGG GAT TGG-3’, Nanog (F) 5’-AGA AGG CCT CAG CAC CTA-3’, Nanog (R) 5’-GGC CTG ATT GTT CCA GGA TT-3’; Twist (F) 5’-CTC GGA CAA GCT GAG CAA GAT TCA GA-3’, Twist (R) 5’-CGT GAG CCA CAT AGC TGC AGC-3’, Slug (F) 5’- ACA CAT TAC CTT GTG TTT GCA AGA TCT-3’, Slug (R) 5’- TGT CTG CAA ATG CTC TGT TGC AGT G-3’, Snail (F) 5’- CCT CAA GAT GCA CAT CCG AAG CCA C-3’, Snail (R) 5’- CCG GAC ATG GCC TTG TAG CAG C-3’, GAPDH (F) 5’-GGT GGT CTC CTC TGA CTT CAA CA-3’, GAPDH (R) 5’-GTT GCT GTA GCC AAA TTC GTT GT-3’.

### Hoechst 33342 dye efflux assay for SP analysis and cell sorting

Adherent cells were harvested using accutase reagent (Sigma Inc). Human Tumor tissue grown in athymic nude mice was minced, enzymatically digested with 0.2% collagenase IV (Worthington Biochemical Corporation) prepared in 10% FBS containing medium for 60 min at 37°C. The digest was further disaggregated by passing through 10 ml pipette several times and filtered through 100/70-μm cell strainer to obtain a single cell suspension. Cells were washed and resuspended in HBSS at 1X10^6^ cells/ml density and incubated with 4 μg/ml of Hoechst 33342 dye (Invitrogen) for 90 min at 37^0^C in presence or absence of 1 μM FTC, as described by Goodell et al. [21]. Cells were incubated with 2 μg/ml Propidium iodide (PI; Sigma Inc) before analysis to visualize and exclude the non-viable cells. The Hoechst 33342 dye was excited at 350 nm using UV laser and its fluorescence was analyzed using 400–500 nm BP filter for blue emission and 640–680 nm BP filter in combination with 655 nm LP-filter for red emission. Flow cytometers from BD Biosciences were used for data acquisition. Data were acquired using LSRII or FACS Vantage (DiVa), and sorted using FACS Vantage (DiVa) cell sorter. Data analyses were done using FlowJo software (Tree Star). Cell cycle analyses for fixed cells were performed for PI stained cells using Vindelov method with similar protocol as described earlier [41].

### Sphere formation or Self renewal assay

Sorted SP or MP cells were plated in 96 well plates at the density of 10,000 cells/ml (1000 cells/well in 100 μl medium) in serum free stem cell selective media (DMEM/F12K (1:1) (Invitrogen), supplemented with 1X-N2 supplement (Invitrogen), 10 ng/ml EGF and 10 ng/ml bFGF (Sigma)) and allowed to grow as spheres for 10 days. Images of the spheres were taken using phase contrast microscope (Nikon) and total numbers were counted. To study the effect of drugs on the self-renewal of SP cells, drugs were added to the respective wells on day 1 and 5 and size and number of the spheres were analyzed on day 10.

### Immunofluorescence

For immunostaining, spheres were transferred to poly D-lysine/Laminin coated glass surface for 18 h. For monolayer cultures, cells were directly plated over the poly D-lysin/Laminin coated glass surface and cultured or treated in stem cell selective media as indicated. Immunofluorescence staining was performed as described previously [42]. Cells were observed using a Leica TCS SP5 confocal microscope (Leica Microsystems) at × 630 magnification.

### Immunohistochemistry

Human lung cancer tissue microarray (TMA) slides with stage I/II or stage IV NSCLC patients were obtained through Lung Cancer Specialized Program of Research Excellence (SPORE). TMA slide with stage I/II tumor samples contained usable cores from 193 patients, and TMA slide with stage IV tumor samples contained usable cores from 103 patients including 17 adenocarcinoma samples from the metastatic sites. The Immunohistochemical staining was performed as described [42]. The samples were scored by a pathologist (D. Coppola). The semiquantitative score was reached by taking into consideration both cellularity and intensity of expression (semiquantitative score = cellularity × intensity). Cellularity was scored as follows: a score of 3 equals to greater than 66% cellularity, a score of 2 equals to 34%–65% cellularity, and a score of 1 equals to less than 33% cellularity. Intensity was scored as follows: a score of 3 equals to strong intensity, a score of 2 equals to moderate intensity, and a score of 1 equals to weak intensity [42]. The score of 1 or above was considered as positive expression of Sox2. The images were captured at × 200 magnification.

### *In vivo* tumor formation assay and bioluminescence imaging

5-weeks-old female SCID-beige mice were used for these experiments under an IACUC approved protocol. For orthotopic implantation of tumor cells, sorted SP or MP cells from A549 cell line stably expressing luciferase gene (A549-Luc) were washed with serum-free DMEM-F12K medium and resuspended at indicated numbers in HBSS containing 500 μg/ml growth factor reduced Matrigel. Surgical procedure for orthotopic lung implantation was followed as suggested earlier for intrapulmonary implantation of tumor cells with some modifications [43]. Specifically, cells were inoculated with 1 ml syringes with 30-gauge hypodermic needles in an open technique under direct visualization into the right lung tissue of SCID mice anesthetized by gas anesthesia (3% isoflurane). Tumor growth/metastases were imaged weekly using bioluminescence by IVIS-200 imaging system from Caliper Corporation. Mice were anesthetized and 30 mg/Kg of D-luciferin in PBS was administered by intraperitoneal (i.p.) injection. Ten minutes after injection, bioluminescence was imaged with a charge-coupled device camera (Caliper) with an imaging time of 2 min. At the end of the experiment, or when mice become moribund, all of the mice were euthanized and individual organs harvested for evaluation of tumor size; distant metastases was determined by bioluminescence of luciferase expressing cells.

### Statistical methods

Data were presented as the mean ± standard deviation (SD). To assess the statistical significance of differences, student’s *t* test was performed. The data were considered statistically significant when the *P* value was less than 0.05.

## Competing interest

We do not have any conflict of interest.

## Authors’ contributions

SS conducted the experiments and wrote the initial version of the manuscript; JT and NBS conducted certain experiments; DC did pathological analysis of the samples; EH provided intellectual input; SA provided human tumor xenografts and input; SC directed the project and finalized the manuscript. All authors read and approved the final manuscript.

## Supplementary Material

Additional file 1 **Figure S1.** BIBW2992 inhibits EGFR phophorylation. H1975 cells were treated with 500 nM gefitinib or 200 nM BIBW2992 for 5 days. EGFR phosphorylation and total EGFR expression was detected in presence or absence of drug treatment. **Figure S2**. Downregulation of Sox2 expression after EGFR and Src inhibition. H1650-SPAdh cells were treated plated over PDL-Laminin coated glass surface and treated with indicated drugs for 4 days. (A) Expression of Sox2 was monitored by immunofluorescence confocal imaging. Isotype antibody was used to show the specific staining of Sox2. (B) Number of Sox2 positive cells for each treatment condition were converted into percentage and plotted. P values were calculated from three different experiments and suggested a significant decrease in Sox2 positive cells after EGFR and Src inhibition. (C) Under similar treatm,ent conditions cells were stained with Nanog specific antibodies. Drug treatment did not alter the expression of Nanog in H1650-SPAdh cells. **Figure S3**. Depletion of Sox2 expression suppresses SP frequency. (A) A549, H1650 and H1975 cells were transiently transfected with second set siRNA (purchased from Origene). 48 h after transfection, cells were analyzed for SP frequency. Similar to first set of siRNA (purchased from SantaCruz), depletion of Sox2 resulted in significant decrease in SP frequency in NSCLCs. (B) NSCLC cells were transfected with Sox2 SIRNA and ABCG2 expression was detected by western blotting. β-Actin was used as internal control for equal loading. * p<0.05. Click here for file
