# Supplementary material for: EGFR/Src/Akt signaling modulates Sox2 expression and self-renewal of stem-like side-population cells in non-small cell lung cancer
Source: Mol Cancer. 2012 Sep 25;11:73. doi: 10.1186/1476-4598-11-73 (PMC3497614; doi:10.1186/1476-4598-11-73)
Supplement: Additional file 1 — Figure S1. BIBW2992 inhibits EGFR phophorylation. H1975 cells were treated with 500 nM gefitinib or 200 nM BIBW2992 for 5 days. EGFR phosphorylation and total EGFR expression was detected in presence or absence of drug treatment. Figure S2. Downregulation of Sox2 expression after EGFR and Src inhibition. H1650-SPAdh cells were treated plated over PDL-Laminin coated glass surface and treated with indicated drugs for 4 days. (A) Expression of Sox2 was monitored by immunofluorescence confocal imaging. Isotype antibody was used to show the specific staining of Sox2. (B) Number of Sox2 positive cells for each treatment condition were converted into percentage and plotted. P values were calculated from three different experiments and suggested a significant decrease in Sox2 positive cells after EGFR and Src inhibition. (C) Under similar treatm,ent conditions cells were stained with Nanog specific antibodies. Drug treatment did not alter the expression of Nanog in H1650-SPAdh cells. Figure S3. Depletion of Sox2 expression suppresses SP frequency. (A) A549, H1650 and H1975 cells were transiently transfected with second set siRNA (purchased from Origene). 48 h after transfection, cells were analyzed for SP frequency. Similar to first set of siRNA (purchased from SantaCruz), depletion of Sox2 resulted in significant decrease in SP frequency in NSCLCs. (B) NSCLC cells were transfected with Sox2 SIRNA and ABCG2 expression was detected by western blotting. β-Actin was used as internal control for equal loading. * p<0.05. [file 1476-4598-11-73-S1.docx]

**Supplementary Figures:**

**
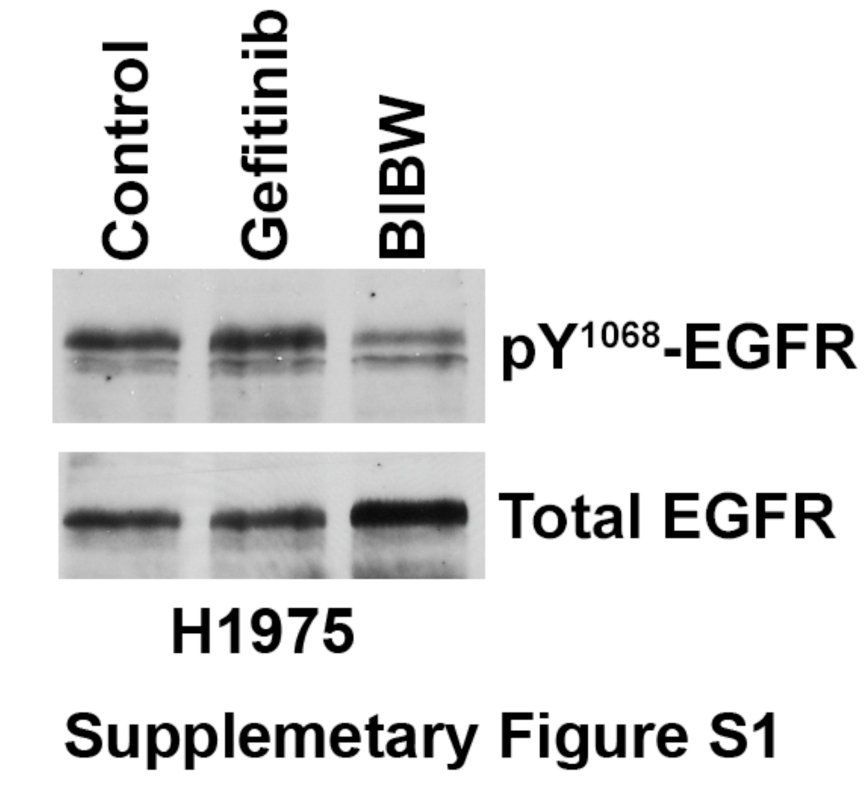
**

**Supplementary Figure S1: BIBW2992 inhibits EGFR phophorylation.** H1975 cells were treated with 500 nM gefitinib or 200 nM BIBW2992 for 5 days. EGFR phosphorylation and total EGFR expression was detected in presence or absence of drug treatment.

**
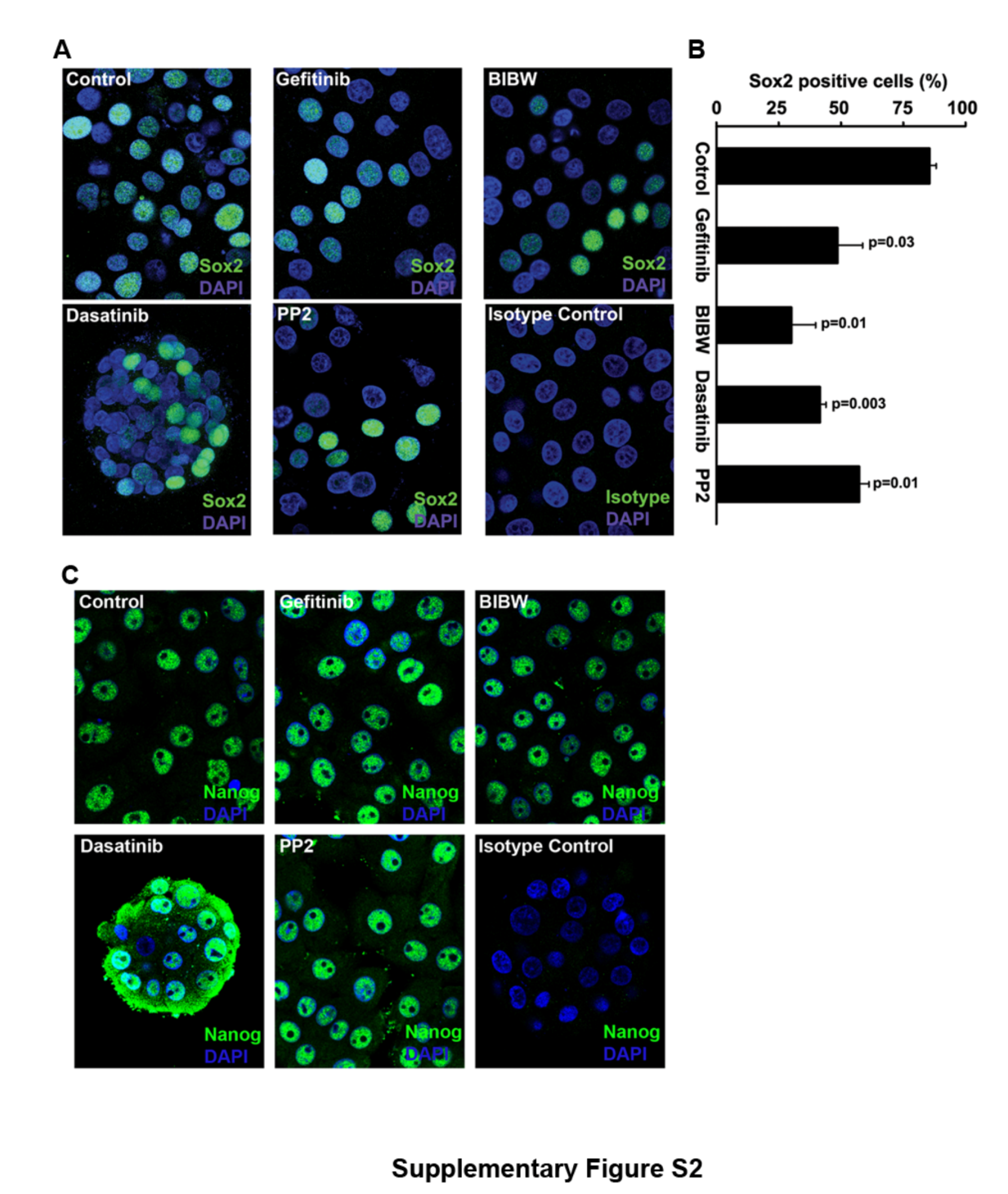
**

**Supplementary Figure S2: Downregulation of Sox2 expression after EGFR and Src inhibition.** H1650-SPAdh cells were treated plated over PDL-Laminin coated glass surface and treated with indicated drugs for 4 days. **(A)** Expression of Sox2 was monitored by immunofluorescence confocal imaging. Isotype antibody was used to show the specific staining of Sox2. **(B)** Number of Sox2 positive cells for each treatment condition were converted into percentage and plotted. *P* values were calculated from three different experiments and suggested a significant decrease in Sox2 positive cells after EGFR and Src inhibition. **(C)** Under similar treatm,ent conditions cells were stained with Nanog specific antibodies. Drug treatment did not alter the expression of Nanog in H1650-SPAdh cells.


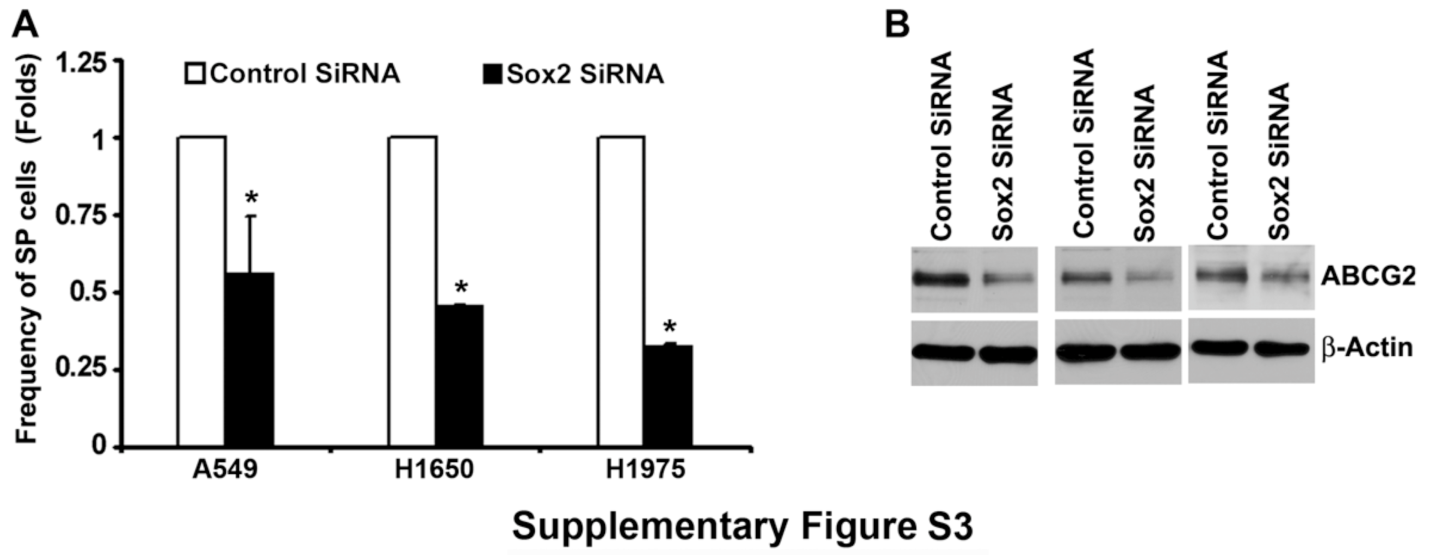


**Supplementary Figure S3: Depletion of Sox2 expression suppresses SP frequency. (A)** A549, H1650 and H1975 cells were transiently transfected with second set siRNA (purchased from Origene). 48 hr after transfection, cells were analyzed for SP frequency. Similar to first set of siRNA (purchased from SantaCruz), depletion of Sox2 resulted in significant decrease in SP frequency in NSCLCs. **(B)** NSCLC cells were transfected with Sox2 SIRNA and ABCG2 expression was detected by western blotting. β-Actin was used as internal control for equal loading. * p<0.05.
